# Single-Center Experience of Focal Thermo-Ablative Therapy After Pelvic Radiotherapy for In-Field Prostate Cancer Oligo-Recurrence

**DOI:** 10.3389/fonc.2021.709779

**Published:** 2021-07-26

**Authors:** Nicolas Giraud, Xavier Buy, Nam-Son Vuong, Richard Gaston, Anne-Laure Cazeau, Vittorio Catena, Jean Palussiere, Guilhem Roubaud, Paul Sargos

**Affiliations:** ^1^ Radiation Oncology Department, Institut Bergonié, Bordeaux, France; ^2^ Oncologic Imaging Department, Institut Bergonié, Bordeaux, France; ^3^ Urology Department, Clinique Saint Augustin, Bordeaux, France; ^4^ Nuclear Medicine Department, Institut Bergonié, Bordeaux, France; ^5^ Medical Oncology Department, Institut Bergonié, Bordeaux, France

**Keywords:** prostate cancer, focal therapy, cryotherapy, oligo-recurrence, interventional radiology, radiation therapy

## Abstract

**Purpose:**

In-field prostate cancer (PCa) oligo-recurrence after pelvic radiotherapy is a challenging situation for which metastasis-directed treatments may be beneficial, but options for focal therapies are scarce.

**Methods:**

We retrospectively reviewed data for patients with three or less in-field oligo-recurrent nodal, bone and/or locally recurrent (prostate, seminal vesicles, or prostatic bed) PCa lesions after radiation therapy, identified with molecular imaging (PET and/or MRI) and treated by focal ablative therapy (cryotherapy or radiofrequency) at the Institut Bergonié between 2012 and 2020. Chosen endpoints were the post-procedure PSA response (partially defined as a >50% reduction, complete as a PSA <0.05 ng/ml), progression-free survival (PFS) defined as either a biochemical relapse (defined as a rise >25% of the Nadir and above 2 ng/ml), radiological relapse (on any imaging technique), decision of treatment modification (hormonotherapy initiation or line change) or death, and tolerance.

**Results:**

Forty-three patients were included. Diagnostic imaging was mostly 18F-Choline positron emission tomography/computerized tomography (PET/CT) (75.0%), prostate specific membrane antigen (PSMA) PET/CT (9.1%) or a combination of pelvic magnetic resonance imaging (MRI), CT, and 99 mTc-bone scintigraphy (11.4%). PSA response was observed in 41.9% patients (partial in 30.3%, complete in 11.6%). In the hormone-sensitive exclusive focal ablation group (n = 31), partial and complete PSA responses were 32.3 and 12.9% respectively. Early local control (absence of visible residual active target) on the post-procedure imaging was achieved with 87.5% success. After a median follow-up of 30 months (IQR 13.3–56.8), the median PFS was 9 months overall (95% CI, 6–17), and 17 months (95% CI, 11–NA) for PSA responders. Complications occurred in 11.4% patients, with only one grade IIIb Dindo–Clavien event (uretral stenosis requiring endoscopic uretrotomy).

**Conclusion:**

In PCa patients showing in-field oligo-recurrence after pelvic radiotherapy, focal ablative treatment is a feasible option, possibly delaying a systemic treatment initiation or modification. These invasive strategies should preferably be performed in expert centers and discussed along other available focal strategies in multi-disciplinary meetings.

## Introduction

Prostate cancer (PCa) ranks among the leading diagnosed cancers and causes of male cancer deaths worldwide, with an estimated 1,276,000 new cancer cases and 359,000 deaths in 2018, a number expected to grow in the upcoming years due to the growth and aging of the population (1). Despite recent advances in the metastatic setting, the number of systemic agents remains limited, especially once castration-resistant status is acquired. The role of local therapies, once restricted to localized disease with curative intent or palliative purposes, is gaining importance and has even shown overall survival benefits when treating newly diagnosed low metastatic burden prostate cancer with radiation therapy (RT) to the prostate in addition to the standard systemic treatment (2, 3).

The concept of oligo-metastatic disease, originating in Hellman and Weichselbaum theories over 20 years ago, showcases growing interest, notably with the development of more accurate imaging modalities, precise focal treatments like stereotactic body radiation therapy (SBRT), and more conservative surgery procedures (4, 5). In PCa, led by advances in terms of imaging with the successive appearance of more sensitive radiotracers in 18F-sodium fluoride (NaF), 18F-Choline, and 68Ga-prostate-specific membrane antigen (PSMA) positron emission tomography/computed tomography (PET/CT), as well as emerging therapies offering prolonged survival, oligo-metastatic disease is increasingly diagnosed. The goal is to treat locally and aggressively every visible location with curative intent for cancers harboring a small number of metastatic lesions, classically less than five, possibly reflecting less aggressive cancers with better prognosis (6). Recently, a few phase II and III trials have shown the benefit of such strategies in terms of progression-free survival (PFS) and overall survival when used as consolidation or in addition to the systemic standard of care, for various neoplasms (7–9).

In PCa, it is theorized that oligo-metastatic evolutions may account for a peculiar form of prostatic disease intermediary between localized and widespread metastatic disease, with slower growth and less aggressive phenotypes, amenable to metastasis-directed therapies (MDTs) (10). One of the goals is to delay the instauration or modification of a systemic treatment (androgen deprivation therapy (ADT) possibly combined with chemotherapy or next generation hormonal therapy), especially for slowly evolving diseases, to avoid toxicities and gain time before the initiation of a new systemic line. This idea is being explored in ongoing or recently presented phase II–III trials, either with purely pelvic oligo-recurrent lesions (excluding patients with history of pelvic RT) in OLIGOPELVIS 2 and STORM (NCT03569241) (11), or distant metastatic oligo-recurrence with the PCS IX (NCT02685397), PRESTO, STOMP, POPSTAR, POSTCARD, and ORIOLE trials (12–16); MDT being mostly SBRT or surgery in these trials.

Nevertheless, in case of in-field relapses after RT, options may be limited and defining the optimal strategy can be challenging. Salvage therapeutic options may include surgery, high-intensity focused ultrasound (HIFU), or re-irradiation (brachytherapy or SBRT). Thermo-ablative procedures are another viable option, historically mostly used for the salvage treatment of locally recurrent PCa, but can also be used to treat nodal or bone lesions especially when not amenable to other MDTs.

In this study, we aimed to retrospectively assess the oncological and toxicity outcomes after thermo-ablative therapy for in-field oligo-recurrent PCa (≤3 bone, nodal and/or locally recurrent lesions) in our French experienced single center.

## Materials and Methods

### Patient Selection and Collected Variables

All patients harboring PCa who received thermo-ablative procedures for metastatic prostate cancer between January 2012 and December 2020 in our center were retrospectively reviewed. Inclusion criteria were patients with oligo-recurrence eligible for MDT to all visible lesions on imaging (PET–CT or a combination of CT/MRI/bone scintigraphy) delivered with curative intent. Patients without history of RT, with lesions not located inside a previous RT field (inside a previous planning target volume with curative intent, according to contemporary recommendations (17–19)), or with non-adenocarcinoma histology were also excluded. Patients treated with a palliative pain-relief objective (palliative treatment in poly-metastatic patients), or with >3 lesions were also excluded. All treatment decisions must had been validated in multidisciplinary concertation meetings. Institutional Review Board approval was obtained.

Variables of interest were extracted from individual patient medical records, including age of patients at the time of procedure, initial PCa characteristics [Gleason score and ISUP group, cTNM, and pTNM in case of surgery, initial prostate serum antigen (PSA), D’Amico risk classification], and previous treatment sequence (surgery, radiation therapy, prior ADT). Limited lymphadenectomy included the obturator chain bilaterally, while extended lymphadenectomy involved bilateral chains: obturator, external-, internal-, common iliac and presacral (20). The latest pre-procedure PSA results were congregated in order to assess the PSA doubling-time (PSA-DT) according to the MSKCC nomogram (21). The number and localization of treated lesions, diagnostic imaging modality as well as the thermo-ablative technique (radiofrequency or cryoablation) and eventual use of concomitant systemic treatment were compiled.

During follow-up, every 3 months for 6 months then every 6 months, acute and late toxicities, dates of biochemical, radiological progressions, and date of death were collected, as well as the date of systemic treatment initiation or modification (including ADT and date of castration-resistant systemic line initiation) and date of new focal treatment. Date of last follow-up was defined as the last available consultation date or date of death.

### Thermo-Ablative Procedures

Procedures were performed by two interventional radiologists with several years of experience in percutaneous thermal ablation of various tumors such as liver, kidney, lung, and musculoskeletal tumors. To offer a better comfort to the patients and as prone position was often required to reach the tumor, general anesthesia was preferred.

**Figure 1:**
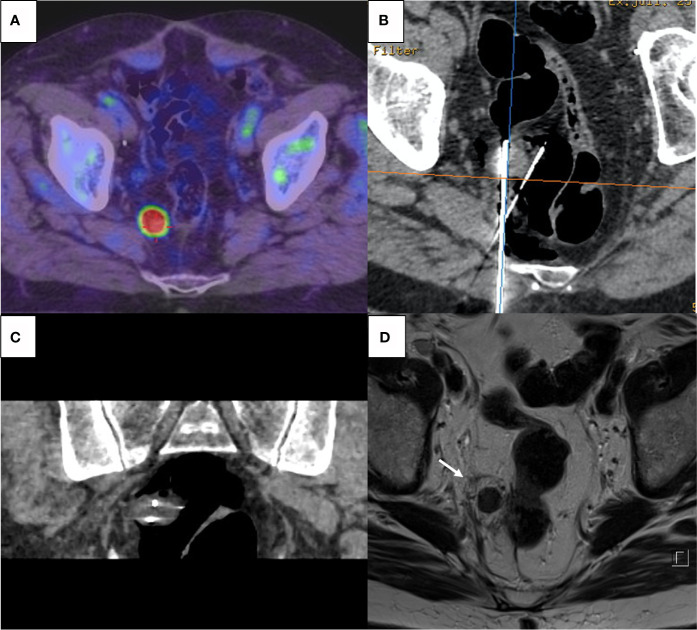
**(A)** Pre-procedure PET-Choline showing a 2 cm hypermetabolic lymph node abutting the rectal wall. **(B, C)** CT-guided percutaneous cryoablation with axial view **(B)** and coronal reconstruction **(C)**; two cryoprobes were inserted into the target and CO_2_ insufflation *via a* 22-gauge spinal needle was performed to achieve rectal wall displacement and isolation. **(D)** T2-weighted MRI after 6 weeks showing complete necrosis of the nodule with surrounding halo of cytosteatonecrosis (arrow); absence of complication on the rectal wall.

Prostatic bed or endopelvic soft tissue ablation was performed with cryoablation, using a last generation argon-based cryoablation machine (Visual Ice, Boston-Scientific, USA). The number and type of cryoprobes (17 gauge IceRod or IceSphere) varied, depending on tumor size and location. All endopelvic procedures were performed under CT guidance to achieve optimal control of the ice ball and surrounding at-risk organs (**Figure 1**). For all interventions, a double 10 min freezing cycle was applied.

Pelvic bone metastases were treated with radiofrequency ablation (Cool-tip, Medtronic, USA) using a single 17 gauge electrode. A 6 to 10 min ablation time was applied to achieve power rolloff. The goal was to reach a temperature plateau >65°C at the tip of the electrode.

Cryoablation was preferred for soft tissue lesions offering a precise control of the ice flow and a real time visualization in these structures to protect highly vulnerable surrounding structures (*e.g.*, sciatic nerve branches or urinary meatus). In opposition, radiofrequency was favored for bone lesions because of a faster procedure, its lower cost, and the lesser concern about surrounding structures as well as a mediocre visualization of the cryoablation ice flow in bone densities. To protect surrounding organs such as rectum, ureters, bladder, or nerves, various thermal protection techniques were used (22). Organ displacement or insulation was performed with hydrodissection or CO_2_ dissection. For tumors abutting major nerves, additional thermocouple was inserted to achieve continuous focal temperature monitoring.

### Outcomes

The primary endpoint was the PSA response, defined as partial (>50% decline from pre-procedure PSA) or complete (PSA <0.05 ng/ml post-procedure) (23, 24). PFS was defined, similarly to the ORIOLE trial (15), as failure occurring during follow-up of either a biochemical progression (PSA increase of >25% of the Nadir and >2 ng/ml), ADT initiation for any reason, clinical or radiological progression [according to RECIST (25)], or death. In our center, ADT initiation was routinely discussed in a multidisciplinary setting and motivated based on the evidence of radiological metastatic evolution after biochemical relapse per the Phoenix guidelines (PSA ≥2 ng/ml + Nadir) or a combination of characteristics according to the European guidelines (PSA-DT <6–12 months and Gleason >7, ISUP grade >3) if no metastatic lesion was highlighted (26, 27).

A post-procedure local evaluation (preferably by MRI) was almost systematically performed during the following first months in order to assess the early local control (defined as the absence of visible local residual active disease) and absence of procedure-related complication.

Acute (≤3 months) and distant toxicities during follow-up were also collected and graded according to the Dindo–Clavien classification (28).

### Statistical Analysis

Statistical analyses were performed to compare subgroups using Student’s t-test if applicable or Wilcoxon tests for quantitative variables, and Fisher exact tests for qualitative variables.

Log rank tests and Kaplan–Meier curves were used to assess survival outcomes. P-value of <.05 was considered significant. All statistical tests were computed using RStudio (v1.3.959).

## Results

### Characteristics of Patients

**Figure 2:**
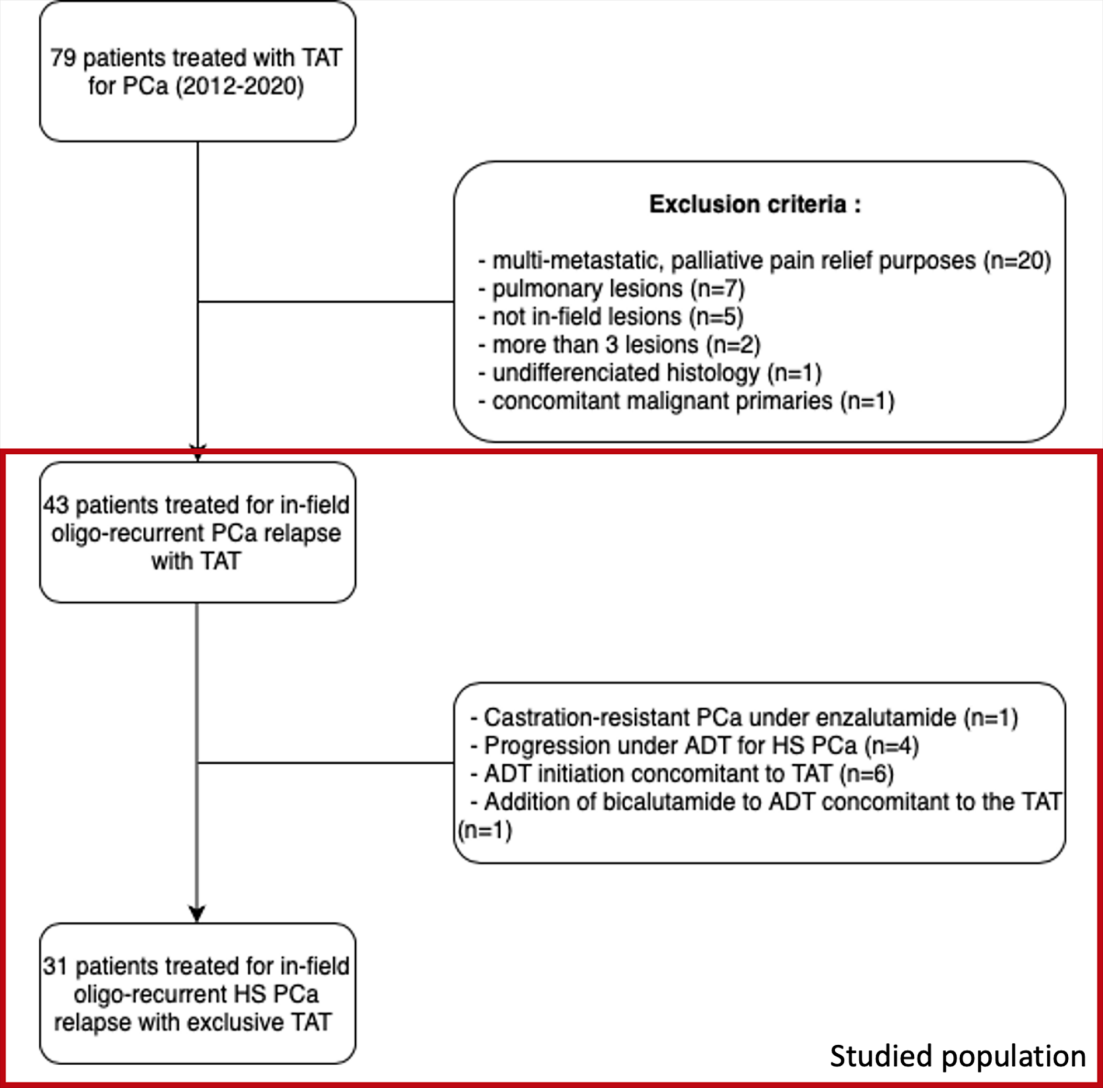
Flowchart of patient selection. TAT, thermo-ablative therapy, PCa, prostate cancer, ADT, androgen deprivation therapy, HS, hormone-sensitive.

Between January 2012 and December 2020, 105 focal thermo-ablative procedures (cryoablation or radiofrequency) were performed for 79 patients with PCa in our center. Among these 79 patients, twenty were treated for palliative purposes (pain relief of bone metastases), seven for extra-pelvic lesions and were thus excluded. Nine other patients were excluded for various reasons (no pelvic RT or lesions outside the previous field of treatment for five patients, concomitant bladder and prostate primaries for one patient, more than three lesions for two patients, undifferentiated histology for one patient). Detailed flowchart can be visualized in **Figure 2**.

In the end, our cohort consisted of 43 patients treated for in-field pelvic oligo-recurrent relapses, with a total number of 49 lesions treated. To be noted is that one patient had three nodal lesions, one in the pelvis treated by cryotherapy and two others outside the previous radiation fields treated by RT. Baseline characteristics of these 43 patients are presented in **Table 1**.

**Table 1:** Baseline patient characteristics among prostate cancer patients treated by thermo-ablative therapy for in-field oligo-recurrence.

	Overall cohort (n = 43)
Age at procedure (mean [extremes], years) (n = 43)	72 [51;86]
Gleason score (n = 42)	
5	1 (2.4%)
6	4 (9.6%)
7	24 (57.1%)
3 + 4	13 (30.9%)
4 + 3	11 (26.2%)
8	8 (19.0%)
3 + 5	2 (4.7%)
4 + 4	6 (14.3%)
9	5 (11.9%)
ISUP score (n = 42)	
1	5 (11.9%)
2	13 (31.0%)
3	11 (26.1%)
4	6 (14.3%)
5	7 (16.7%)
Initial PSA [mean (extremes), ng/ml] (n = 32)	16.9 [3.86;129]
Initial T stage (n = 42)	
T2a	1 (2.4%)
T2b	6 (14.3%)
T2c	8 (19.0%)
T3a	13 (31.0%)
T3b	14 (33.3%)
Initial N stage (n = 42)	
N0	39 (92.9%)
N1	3 (7.1%)
Initial R stage (in case of surgery) (n = 31)	
R0	23 (74.2%)
R1	8 (25.8%)
Treatment sequence (n = 43)	
Initial prostatectomy + salvage RT	32 (74.4%)
Extended lymphadenectomy	21 (65.6%)
Limited lymphadenectomy	2 (6.3%)
No lymphadenectomy	9 (28.1%)
Initial RT +/− ADT	11 (25.6%)
Previous RT field (n=43)	
Prostate only	1 (2.3%)
Prostate + whole-pelvis	10 (23.3%)
Prostatic bed only	12 (27.9%)
Prostatic bed + whole-pelvis	20 (46.5%)
Last PSA before procedure [mean (extremes), ng/ml] (n = 43)	5.0 [0.3;22.0]
Calculated PSA doubling time [mean (extremes), months] (n = 43)	8.9 [1.4;55.1]
Target maximum diameter [mean (extremes), mm] (n = 43)	15.2 [6;57]
Type of lesion treated (n = 49)	
Bone	13 (26.5%)
Node	15 (30.6%)
Prostate, prostatic bed or seminal vesicles	21 (42.9%)
Concomitant ADT (n = 43)	
No	31 (72.1%)
Yes, started or modified concomitantly	7 (16.3%)
Yes, previously in place	5 (11.6%)
HSPC	4 (9.3%)
HRPC	1 (2.3%)

RT, radiation therapy; ADT, androgen deprivation therapy; PSA, prostate serum antigen; HSPC, hormone-sensitive prostate cancer; HRPC, hormone-resistant prostate cancer.

In this population, one patient was already treated for castration-resistant PCa with enzalutamide, the focal treatment goal aiming to avoid a change of systemic line, considered as an “oligo-progressive disease” (29). Likewise, four patients were undergoing total androgen blockade, the focal approach hoping to postpone a castration-resistant systemic first line. Finally, six patients started short ADT and one already under ADT received the addition of bicalutamide concomitant to the focal treatment.

Considering our ADT-free hormone-sensitive population of 31 patients, the median age was 72 years (range, 51–81), with patients presenting mostly high initial d’Amico risk scores (93.5%). Pre-procedure PSA was 2.48 ng/ml (range, 0.43–14.5) and pre-procedure calculated PSA-DT was 7.6 months (range, 1.4–55.1). Thirty-four lesions were treated by thermo-ablative therapy among which six were pelvic bone lesions (pubic rami, symphysis), twelve were nodal targets and sixteen were (in place or post-surgical) prostate or seminal vesicle recurrences.

### Diagnostic Modality and Ablative Procedure

Diagnostic imaging consisted in PET-Choline for thirty-three patients (76.7%), PET-PSMA for four patients (9.3%), PET-NaF for one patient (2.3%), and a combination of MRI, CT, and bone scintigraphy for five patients (11.6%).

Focal thermo-ablative procedure was RF for 10 lesions (20.4%) and cryotherapy for 39 lesions (79.6%). Only one lesion, situated on the pubic symphysis, received an additional thermo-ablative treatment due to its size and the persistence of visible disease on the post-procedure MRI. Among the overall population, respective median and mean maximum diameters of the treated lesions were 12 and 15.2 mm (range, 6–57); 11 and 12.3 mm (range, 6–35) in the ADT-free cohort.

### Outcomes

Among the overall population of 43 patients, PSA response was observed in 41.9% (n = 18): partial in 30.3% (n = 13) and complete in 11.6% (n = 5) patients. The post-procedure local evaluation was performed in 90.9% of patients (n = 40), with a median delay of 1 month (range, 0–8 months). Early local control was achieved with 87.5% success (35/40 patients), local progression being observed in the treatment of bone lesions for three patients and seminal vesicles for two patients.

**Figure 3:**
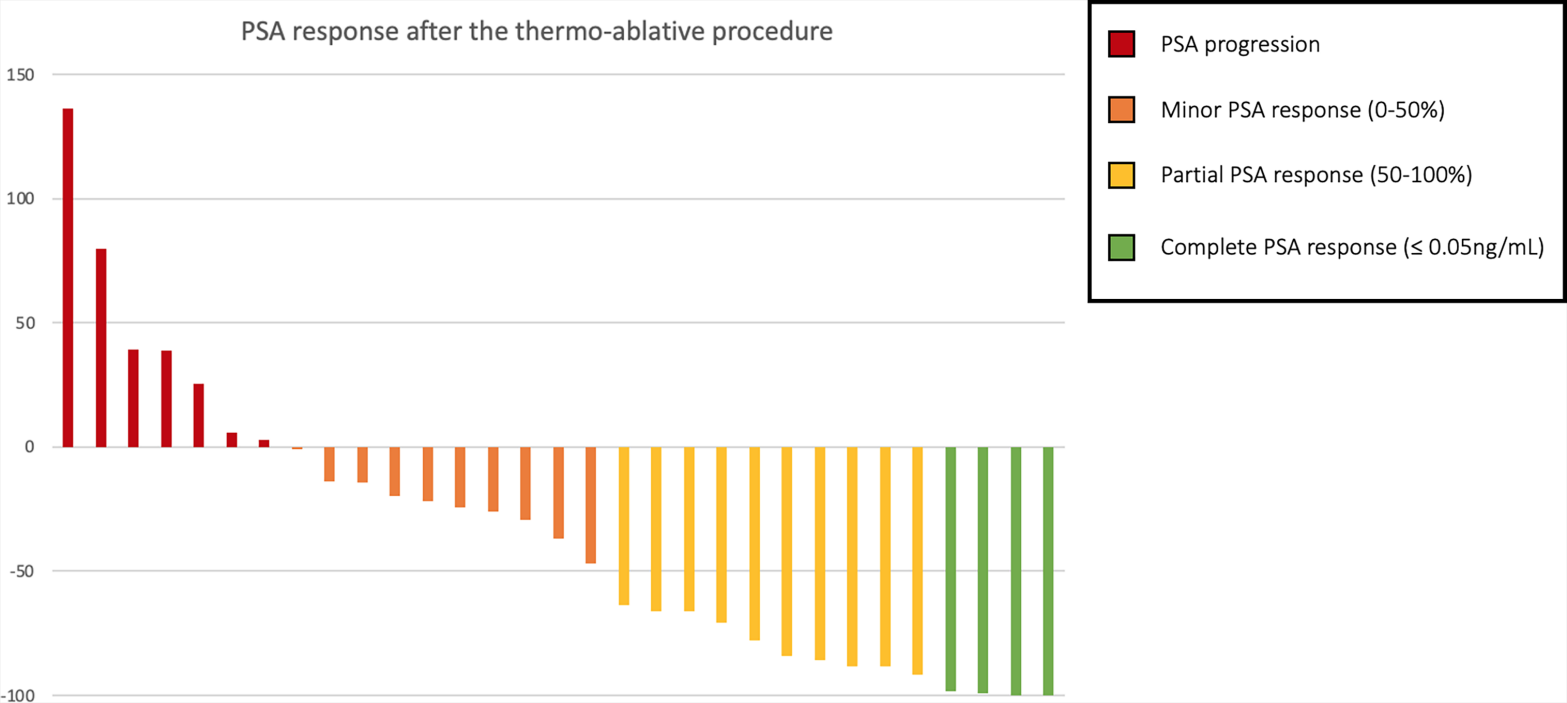
PSA response in percent of pre-procedure PSA among oligo-recurrent hormone-sensitive prostate cancer patients treated with exclusive thermo-ablative therapy.

Considering solely the 31 patients treated in the castration-sensitive setting with exclusive focal therapy (no concomitant ADT), a PSA response was observed in 45.2% patients (n = 14): partial in 32.3% (n = 10), and complete in 12.9% (n = 4). A visual representation of the PSA response can be found in **Figure 3**. With a median follow-up of 30 months (IQR 13.3–56.8), the median PFS was 9 months (95% CI, 6–17), and the median time to initiation of ADT was 11 months (95% CI, 9–47). Three deaths were observed at the end of follow-up (at respectively 30-, 60- and 85-months post-procedure).

**Figure 4:**
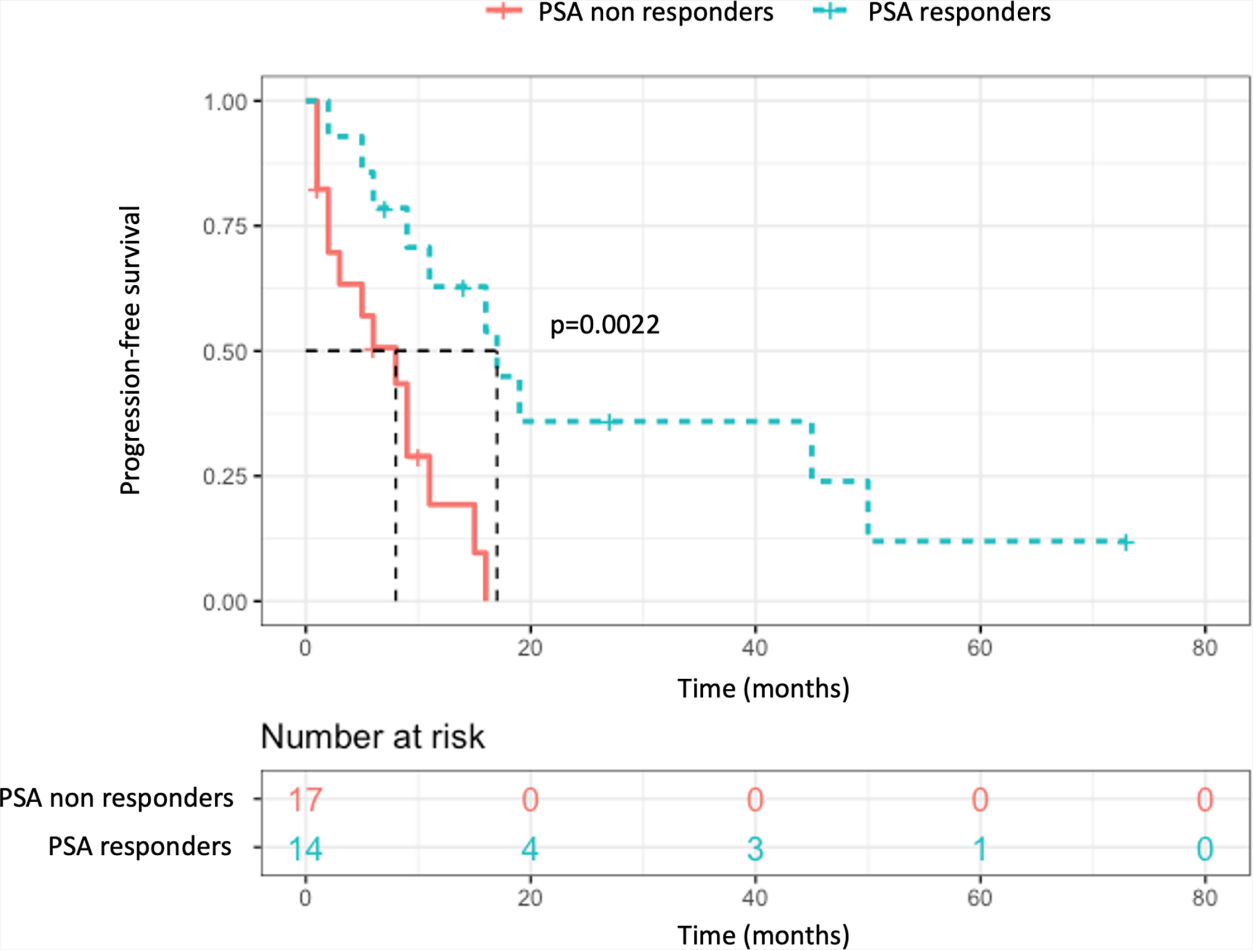
Composite endpoint survival stratified by post-procedure PSA response.

In this population, comparing patients presenting a post-procedure PSA response (at least partial) and those who did not, a significant difference was found regarding the PFS with median survivals of 17 and 8 months respectively (p = 0.002, **Figure 4**), and time to ADT initiation (21 *versus* 8 months, p = 0.014). There was no significant difference found in terms of initial tumoral characteristics, extension, or pre-procedure data, notably regarding the pre-procedure PSA (means of 4.24 and 4.27 ng/ml respectively, p = 0.77) and PSA-DT (10.5 *vs* 9.9 months respectively, p = 0.46) (**Table 2**). Among the four patients who displayed complete PSA response at the first follow-up, prolonged responses were obtained: no event at 7 and 73 months of follow-up for two patients, biochemical relapse at 16 months and 45 months requiring ADT initiation for the two others.

**Table 2:** Statistical comparison of initial and pre-procedure data between PSA responders and non-responders.

	PSA responders	PSA non responders	p-value
Age at procedure (years, mean)	69.1	71.0	0.46
PSA at diagnosis (ng/ml, mean)	14.6	11.5	0.34
Last PSA pre-TAT (ng/ml, mean)	4.24	4.27	0.77
PSA doubling time (months, mean)	10.49	9.85	0.46
Initial Gleason score			0.16
Initial ISUP group			0.45
Initial T-stage			0.15
Initial N-stage			0.48
Initial D’Amico risk group			1
Number of lesions treated			0.77

TAT, thermo-ablative therapy.

Following thermo-ablative therapy, radiological patterns of progression among the 26 patients experiencing biochemical recurrence favored single site recurrences (54%). Multisite relapse (≥5 lesions) was observed in 10%. In patients treated for bone lesions, the vast majority (83%) experienced recurrences that included an osseous site, and for relapses after treating nodal sites, 71.4% occurred exclusively to nodal sites, and 28.6% showcased osseous lesions. Local relapses were observed in six patients (19.4%), mostly treated on the prostate post-operative bed (66.7%). Finally, in five cases, no radiological target could be identified despite reaching biochemical levels of recurrence.

### Toxicity Assessment

Six toxicity events were observed in five patients (11.6%) classified according to the Dindo–Clavien classification: four grade I (acute urinary tract burning sensation, acute dysuria for one week, acute post-operative local pain, prolonged S1 sciatalgia), one grade IIIa (acute urinary retention due to an uretral stenosis requiring a catheter), and one grade IIIb event (urinary incontinence and uretral stenosis requiring endoscopic uretrotomy 16 months after a cryotherapy procedure for a local prostatic recurrence post-RT). Both grade III events occurred after procedures targeting the prostate, with per-operative difficulties due to local considerations (small prostatic gland, fibrosis due to previous RT +/− high-intensity focused ultrasound treatments) and already harboring pre-procedure obstructive urinary symptoms. We did not identify any post-operative urinary or digestive fistula.

## Discussion

In our study, we showed that thermo-ablative therapies were safe, with around 45% PSA responses when treating in-field ADT-free oligo-recurrent PCa, allowing a median of 21-month systemic treatment deferral for PSA responders.

Patterns of PCa recurrence after initial local therapy have been shown to be associated with prostatic cancer-specific survival (PCSS). In 2,694 patients treated with prostate-only RT, prostate was the most common first recurrence site in the low, intermediate, and high risk groups with an 8-year cumulative incidence of 3.5, 9.8, and 14.6% respectively. Moreover, in the 474 patients with clinically detected recurrence, the most common first recurrence site was local in 55.3%, bone in 33.5%, pelvic lymph nodes in 21.3%, and abdominal lymph nodes in 9.1% (30). Likewise, in 574 men treated by salvage RT (SRT) after prostatectomy between 1986 and 2013, the 8-year rates of local, regional, and distant failure were 2, 6, and 21% respectively, of which 17% were lymphotrophic, 50% osteotrophic, and 31% multifocal, a repartition prognostic for distant metastases-free survival and PCSS (31). In our cohort, similarly to Deek et al. (32), patterns of recurrence after MDT were mostly oligo-progressions, with only three patients showing multisite recurrence of ≥5 metastases. Recurrence tended to occur in osseous sites after treating bone targets, and nodal or bone locations when treating patients with nodal-only dissemination.

In case of local relapse after RT, a meta-analysis was recently published including 150 studies regarding outcomes of local therapies. Adjusted 5-year recurrence-free survival ranged from 50% after cryotherapy to 60% after high-dose-rate brachytherapy and SBRT, with no significant differences between any modality and radical prostatectomy. Severe GU toxicity was however significantly lower with salvage RT or cryotherapy than with salvage RP (33). Data for salvage prostate SBRT re-irradiation are emerging but the technique must be administered with caution in expert centers and highly selected patients, with 2- and 3-year disease-free survival rates ranging from 40 to 82% among 38 studies; inclusion in clinical trials is recommended (34).

Thanks to the advent of new radiotracers like Choline or PSMA, oligo-metastatic states (either synchronous or metachronous) are increasingly discovered. In 9,632 restaging Choline PET/CT performed between 2007 and 2015 for biochemical relapse post-RP or RT, Graziani et al. found an incidence of 37.7% of oligometastatic disease defined as one to three lesions (35). These new imaging modalities are especially effective for PSA rates <20 ng/ml compared to the traditional triquetra of CT, bone scintigraphy and MRI. Although Choline-PET already offers good detection, its sensitivity is highly dependent on PSA levels and kinetics (36). PSMA-PET offers even better sensitivity, with more detected lesions in low PSA patients and offering higher contrast with the background noise (37, 38). This is raising new discussions, as this ability to detect and potentially treat locally a few small hypermetabolic lesions questions its place in the oncological strategy. It is hypothesized that local treatments might reduce the number of circulating tumor cells, which were shown to be associated with an increased risk of progression and mortality (39).

Several ongoing or recently published trials explore outcomes of MDT in this oligo-metastatic setting, either nodal pelvic or distant metastatic. For instance, in the phase II STOMP trial, MDT to up to three extracranial oligo-recurrent lesions visible on Choline-PET tended to increase the ADT-free survival compared to surveillance (8 *vs* 34% at 5 years, p = 0.06) (12). Nevertheless, there is significant heterogeneity between local treatment protocols (SBRT fractionation and dose, surgical procedure) and diagnostic imaging between these trials. Inclusions can also be difficult, mostly due to an unwillingness to be assigned in a treatment arm based on randomization (40).

A limited number of papers have been published about in-field MDT after a prior RT, and series comprising re-irradiated patients often intertwine with RT-naïve patients (23, 41). In our cohort of hormone-sensitive PCa oligo-recurrent patients, PSA response was obtained in 45.2% of patients without concomitant ADT, postponing in responders with a median delay of 17 months the need of ADT initiation, disease recurrence or death (9 months in the overall population).

This MDT strategy could also be discussed in an intensification strategy concomitant to ADT. For instance, Kroeze et al. found in 305 PSMA PET-positive oligo-recurrent patients that MDT + ADT significantly improved the biological PFS (hazard ratio 0.28, 95% confidence interval 0.16–0.51), but was not significantly different between MDT and ≤6 months of ADT or MDT alone (p = 0.121). However, the risk of disease progression was higher after local radiotherapy alone without immediate ADT (42) and consequently the need for further treatments. Combination with novel agents like durvalumab, an anti-PD-L1, may also bear promises, like in the ongoing POSTCARD trial (NCT03795207).

Finally, MDT could also be debated for castration-resistant PCa, even if our population was limited in this regard (43). For example, in a prospective study of 29 patients (of which 37.9% were castration-resistant) with oligo-metastatic PCa, SBRT allowed controlled PSA levels in 20 patients (with a median follow-up of 11.5 months), thus avoiding the use of systemic therapy and delayed its use by a median of 39.7 months in the remaining nine patients (44). In this castration-resistant setting a few trials are underway, like the FORCE trial (NCT03556904) for oligo-metastatic patients or the STEREO-RE-PRO trial (NCT03438552) now in phase I to determine the optimal SBRT fractionation before entering phase II aiming to estimate the efficacy of repeat salvage SBRT in terms of biochemical relapse-free survival rate for intraprostatic tumor recurrence.

The choice of focal treatment modality in our center was mainly motivated by the high experience of our interventional radiologists, and the easy access to thermo-ablative techniques, compared to SBRT which was not available at the time in our institution. Selecting the optimal technique should be discussed on a patient-basis, as well as according to local habits.

In our cohort, tolerance was acceptable with only two grade ≥III events according to Dindo–Clavien, both in patients treated on the prostate with prior obstructive urinary symptoms and locally contributing factors. Risks are mainly dependent on the treated target: nervous and vascular lesions for extra-prostatic procedures (especially lymph nodes, with a risk of obturator and sciatic nerves injury when dealing with lesions close to the obturator ring/presacral spaces); genito-urinary and sexual complications in case of local relapse, which is similarly reported in the available literature (0–31% rates of erectile dysfunction, 1–17% rates of urinary retention, and less than 5% rates of urinary incontinence) (45–48).

Our study holds several limitations mostly due to its retrospective nature and the limited size of our cohort emanating from a single institution and containing heterogeneous patients in terms of initial tumor characteristics, natural history, and previous treatments. In the final analysis, only ADT-free hormone-sensitive patients were described, as concomitant systemic treatment would have affected the outcomes. Due to the extended period of study, there is also a discrepancy regarding imaging modalities, notably with the recent advent of Choline and PSMA tracers, which could have impacted the therapeutic strategy.

In the end, patient selection was crucial to sort out which patients could benefit the most from these aggressive strategies. Undergoing trials should provide an answer whether focal therapy is beneficial when treating in-field oligo recurrent patients and eventually translates to time-spared, or time-wasted. In our cohort, no factor (initial patient and tumor’s characteristics or pre-procedure PSA rates and kinetics) was found to be associated with the PSA response. Associating the PSA-DT, biopsy Gleason score and interval from primary therapy to biochemical failure could further stratify patients according to recent recommendations, notably to select patients benefiting from early ADT initiation after non-metastatic PCa relapse (49, 50). In this matter, advances in PCa genomics or even radiomics and artificial intelligence could also generate new hopes with regard to personalized medicine (51–53). Tumor mutational profiles, such as driver mutations in TP53 or alterations in other tumor suppressor genes, could be associated with disparate outcomes among oligo-metastatic PCa, possibly identifying in the near future patients with aggressive features who may benefit from intensified treatment (54–56).

## Conclusion

Thermo-ablative procedures for in-field oligo-recurrent PCa are a feasible option in terms of local control and biochemical response, possibly allowing systemic treatment deferral for patients with in-field oligo-recurring PCa or even potentiating its effects. Patient selection is crucial and could benefit from advances in imaging and prognostic markers. Given the risk of morbidity and need of technical experience, these procedures should be discussed on a case-by-case basis in a multidisciplinary setting and preferably performed in expert centers.

## Data Availability Statement

The raw data supporting the conclusions of this article can be made available by the authors, upon request.

## Ethics Statement

The studies involving human participants were reviewed and approved by Collège de Recherche, Institut Bergonié (April 12, 2021). Written informed consent for participation was not required for this study in accordance with the national legislation and the institutional requirements.

## Author Contributions

NG and N-SV: data collection. NG: data processing and statistical analysis, and original draft writing. XB, RG, VC, JP, GR, and PS: data collection. XB, GR, and PS: supervision. NG, XB, N-SV, RG, A-LC, VC, JP, GR, and PS: manuscript correction. All authors contributed to the article and approved the submitted version.

## Conflict of Interest

The authors declare that the research was conducted in the absence of any commercial or financial relationships that could be construed as a potential conflict of interest.

## Publisher’s Note

All claims expressed in this article are solely those of the authors and do not necessarily represent those of their affiliated organizations, or those of the publisher, the editors and the reviewers. Any product that may be evaluated in this article, or claim that may be made by its manufacturer, is not guaranteed or endorsed by the publisher.
